# Removing inactive NRTIs in a salvage regimen is safe, maintains virological suppression and reduces treatment costs: 96 weeks post VERITAS study

**DOI:** 10.7448/IAS.17.4.19815

**Published:** 2014-11-02

**Authors:** Benoit Trottier, Daniele Longpré, Harold Dion, Sylvie Vezina, Stephane Lavoie, Chrissi Galanakis, Nima Machouf, Réjean Thomas

**Affiliations:** 1 Clinical Research, Clinique Médicale l'Actuel, Montreal, Canada; 2 Department of Medicine, Clinique Médicale l'Actuel, Montreal, Canada; 3 Epidemiology, Clinique Médicale l'Actuel, Montreal, Canada; 4 Directorate, Clinique Médicale l'Actuel, Montreal, Canada

## Abstract

**Introduction:**

In HIV+ patients exhibiting multidrug resistance (MDR), NRTIs often have little activity, increased toxicity, drug interactions and add unnecessary treatment costs. The 48 week VERITAS study demonstrated that these patients can have a safe and effective simplification of salvage regimen by removing inactive NRTIs as determined by genotypic data. Virological, immunological, clinical and financial outcomes were evaluated at an additional 96 weeks of follow-up.

**Materials and Methods:**

MDR patients with an undetectable viral load (VL) on a stable regimen containing at least four ARVs (including one inactive NRTI) were enrolled in an open-label, prospective simplification trial, where one inactive NRTI was removed at baseline (BL). A second NRTI could be removed at week 24 if the regimen contained at least five ARVs at enrolment.

**Results:**

31 male patients participated. The mean length of treatment was 14 years, with a median CD4 count of 525. The BL regimen consisted of 4 ARVs in 22 patients (71%) and 5 ARVs in 9 patients (29%). 3TC or FTC was removed in 29 patients (94%), and either AZT or TDF was interrupted in 2 others. Four patients had a second NRTI stopped. One patient was removed at W26 as an active NRTI was removed for creatinine elevation. 30 well-controlled patients continued follow-up after W48. At W144, six patients had additional changes in their ARV regimen. Half were due to toxicity (jaundice, neuropathy and nephrotoxicity) while the other half were the result of treatment simplification. None of the patients exhibited virologic failure at the time of treatment change and maintained undetectable VLs throughout the entire follow-up. These six patients had a mean gain of 79 CD4 (p=0.17) compared to baseline. 22 of the 24 patients (92%) with no changes in ARV therapy after W48 had undetectable VLs. The other two had confirmed virologic failure, one with genotypic resistance. All 24 had elevated CD4 counts (mean +118 CD4, p<0.0001). No deaths or serious adverse events were observed. One or two ARV removals translated to a mean annual saving of $3319 CDN (11%) and $8630 (24%) respectively.

**Conclusions:**

Final results indicate that removing one or two inactive NRTIs from a regimen in patients taking four or more ARVs with controlled VL appears to be safe, maintains virological suppression through 144 weeks and significantly reduces treatment costs.

